# A Pandemic within Other Pandemics. When a Multiple Infection of a Host Occurs: SARS-CoV-2, HIV and *Mycobacterium tuberculosis*

**DOI:** 10.3390/v13050931

**Published:** 2021-05-17

**Authors:** Carmen María González-Domenech, Isabel Pérez-Hernández, Cristina Gómez-Ayerbe, Isabel Viciana Ramos, Rosario Palacios-Muñoz, Jesús Santos

**Affiliations:** 1 Clinical Research in HIV Infection, Endovascular Infection and Bacteriemia, Biomedical Research Institute of Malaga (IBIMA), 29010 Malaga, Spain; cgayerbe@gmail.com (C.G.-A.); isaviciana@hotmail.com (I.V.R.); rosariopalaci@gmail.com (R.P.-M.); med000854@gmail.com (J.S.); 2 Department of Microbiology, Faculty of Sciences, University of Malaga, 29071 Malaga, Spain; 3 Internal Medicine Service, Melilla Regional Hospital, 52005 Melilla, Spain; isaperezhernandez@gmail.com; 4 Infectious Diseases and Clinical Microbiology Unit, Virgen de la Victoria Hospital, 29010 Malaga, Spain

**Keywords:** SARS-CoV-2, COVID-19, HIV, tuberculosis, coinfection, triple-infection

## Abstract

By the middle of 2021, we are still immersed in the coronavirus disease 2019 (COVID-19) pandemic, caused by the severe acute respiratory syndrome coronavirus 2 (SARS-CoV-2). The concurrence of this new pandemic in regions where human immunodeficiency virus (HIV) and tuberculosis (TB) infections possess the same epidemiological consideration, has arisen concerns about the prognosis, clinical management, symptomatology, and treatment of patients with triple infection. At the same time, healthcare services previously devoted to diagnosis and treatment of TB and HIV are being jeopardized by the urgent need of resources and attention for COVID-19 patients. The aim of this review was to collect any article considering the three conditions (HIV, TB, and SARS-CoV-2), included in PubMed/Medline and published in the English language since the beginning of the COVID-19 pandemic. We focused on detailed descriptions of the unusual cases describing the three co-infections. Eighty-four out of 184 publications retrieved met our inclusion criteria, but only three of them reported cases (five in total) with the three concomitant infections. The clinical evolution, management, and therapy of all of them were not different from mild/severe cases with exclusive COVID-19; the outcome was not worse either, with recovery for the five patients. Cases of patients with COVID-19 besides HIV and TB infections are scarce in literature, but studies deliberately embracing the triple infection as a priori inclusion criterion should be carried out in order to provide a complete understanding of joint influence.

## 1. Introduction

According to UNAIDS global human immunodeficiency virus (HIV) statistics, there were over 38 million people worldwide living with HIV (PLWHIV) at the end of 2019. Most of cases are concentrated in sub-Saharan Africa; among the most devastated countries by HIV epidemic, South Africa possesses the highest number of PLWHIV [1]. Untreated HIV replication causes a wide range of immunological dysfunction, with a progressive loss of CD4(+) T cell and B cell functionality, leading to an increased risk of opportunistic infections and carcinogenic events [2,3,4]. In this context, pneumonia by different pathogens was a leading cause of morbidity and mortality before the institution of antiretroviral therapy (ART) [5]. Afterwards, the implementation of ART from the mid-1990s substantially improved HIV patients’ quality of life and life expectancy to such an extent that they are currently close to those for individuals without HIV infection [6,7,8]. However, far from being an eradication treatment, ART must currently be taken for life. As well-known and presently unsolved, there exists a main obstacle for a functional or sterilizing cure of the HIV infection: its viral latency and ability to rebound viremia following ART interruption. HIV possesses plenty of molecular mechanisms targeted to establish and maintain a latent infection, and, as a consequence, a chronic immune disorder (for a review see [9,10,11]). Besides the effects of HIV-induced persistent systemic inflammation and the impact of long-term ART on respiratory immune response, the lung is a known reservoir for HIV, so this organ may be exposed to an increased frequency of other pulmonary diseases [12].

For its part, tuberculosis (TB) is an ancient but still present human disease caused by *Mycobacterium tuberculosis* (MTB), and almost exclusively transmitted through the air [13,14]. Worldwide, TB was considered in the global top 10 causes of death at the beginning of this century, falling from 7th place in 2000 to 13th in 2019, with a 30% reduction in global deaths. Nevertheless, it remains among the top 10 causes of death in the African and South-East Asian regions. According to World Health Organization (WHO), over 10 million people fell ill with TB worldwide, and almost one million and a half people died from TB (including 208,000 people with HIV) in 2019. About half of the new TB cases occurred in the South-East Asian region (44%), followed by the African region (particularly in sub-Saharan Africa), with a quarter new cases (25%), and the Western Pacific with approximately a fifth (18%), according to Global Health Observatory data repository (2020). Two thirds of the number of cases are concentrated in just eight countries, with India leading the count, followed by Indonesia, China, the Philippines, Pakistan, Nigeria, Bangladesh and South Africa [15]. Pulmonary presentation is the most common, although the mycobacteria can spread to other organs or systems (such as skin, liver, central nervous, musculoskeletal and reproductive systems) through the hematogenous route [16,17]. Somehow similar to a treatment-controlled HIV patient, with the virus concealed itself in CD4+ T cells, there is a latent TB infection characterized by the pathogen lying dormant inside the lungs, without causing destruction of organs [13,14]. Anyway, PLWVIH and also with a latent TB infection present a higher risk of progressing to active disease, with a predominant extrapulmonary manifestation [15,16,18]. Thus, HIV and TB may represent a lethal tandem.

Finally, at the time of writing this review, we are still immersing in the pandemic of coronavirus disease 2019 (COVID-19). The etiological agent is a member of *Coronaviridae* (CoV) family, named severe acute respiratory syndrome coronavirus 2 (SARS-CoV-2) by the International Committee on Taxonomy of Viruses basing on phylogenetic analysis [19]. This family comprises four major genera: *Alphacoronavirus, Betacoronavirus, Gammacoronavirus*, and *Deltacoronavirus*; the two former, the Alpha and Beta-CoVs, have the ability to infect humans [20,21], and cause a range of illnesses, from the common cold to a severe acute respiratory tract infection. The first reported case emerged in Wuhan, Hubei Province, China, in December 2019, and soon turned into a global threat, because of its viral infectivity and its permanent within-host evolution. Since then, the virus has caused over 108 million confirmed cases and almost 2.4 million deaths worldwide at the time of writing (middle of February 2021) [22]. The dominant route of transmission for SARS-CoV-2 is airborne aerosol/droplet, but transplacental transmission is also described [23,24]. After entering the lungs by inhalation, SARS-CoV-2 activates immune system, cytokines, and other pathogen-resistance mechanisms. As previously exposed for HIV, acute infection for SARS-CoV-2 is also associated with lymphopenia, and the severe decline of CD4+ T cell counts and B cell dysfunction in COVID-19 patients have been linked to poor clinical outcomes [25,26,27,28,29,30,31]. The dynamic of lymphocyte subsets changes in the course of both infections are not comparable though, because SARS-CoV-2 chronification is currently ruled out, unlike HIV [32].

In light of the above, the concurrency of a new pandemic within two other pandemics has jeopardized health care providers for TB and HIV patients. Thus, resources achieved over a long period for control of HIV and TB infections are now being redirected to COVID-19. This situation is dramatically evident in many African countries, especially in South Africa [33,34,35,36].

In this review, we collected and summarized any research or scientific thought simultaneously considering these three conditions, especially when concerned impact on public health. We sought studies reporting unusual cases of a triple infection with *Mycobacterium tuberculosis*, HIV, and SARS-CoV-2. Differences and similarities in clinical management, respiratory therapy, symptomatology, or outcomes were searched and compared to those from an exclusive SARS-CoV-2 infection.

## 2. Materials and Methods

A literature search for pertinent peer-reviewed publications was conducted using PubMed/Medline electronic database from December 2019 to 16 February 2021, using the following combination of key terms: [“HIV” AND “TUBERCULOSIS” AND “SARS-CoV-2”], [“AIDS” AND “CORONAVIRUS” AND “TUBERCULOSIS”], and [“HIV” AND “TUBERCULOSIS” AND “Coronavirus”]. The search was limited to publications in English. Inclusion criteria were systematic and narrative reviews, prospective and retrospective studies, case series and case reports considering the concurrence of the three pathogens in a same host. We especially focused on case reports simultaneously assessing any feature of the three infectious diseases: clinical characteristics, epidemiology, diagnosis, care interventions, prognosis, immunology, treatment, or prevention. The most relevant information was inserted in a spreadsheet, in order to make it to easier analyze the data and present the results in a narrative way.

## 3. Results and Discussion

### 3.1. Summary of Literature Search and Selection

We obtain a total of 105 unique records, out of 184 publications retrieved (Supplementary File S1). According to each set of keywords, [“AIDS” AND “CORONAVIRUS” AND “TUBERCULOSIS”], [“HIV” AND “TUBERCULOSIS” AND “SARS-CoV-2”] and [“HIV” AND “TUBERCULOSIS” AND “CORONAVIRUS”], the outcomes were 32, 70 and 82, respectively.

Twenty one out of 105 studies were discarded (Supplementary File S2). Two of them were written in a language other than English; thirteen articles left out a mention to any of the three infections, because some of them were done prior to COVID-19 pandemic; other four papers were not considered because co-infection with HIV and/or Mycobacterium tuberculosis was actually an exclusion criterion in such studies; another case report was discarded since patient had a negative HIV serology, as found out when reading the manuscript.

Thus, 84 studies finally met our inclusion criteria (Supplementary File S3), three of them reporting a detailed description of cases with the three concomitant infections: HIV, TB, and SARS-CoV-2 (Supplementary File S4) [37,38,39].

### 3.2. Case Reports of Triple Infection with HIV, TB and SARS-CoV-2

The detailed description of the five cases is depicted in Supplementary File S4, with their main characteristics shown in Table 1. Four out of the total five ones were published in American Journal of Tropical Medicine and Hygiene [38,39]. The cases reported were from Brazil (*n* = 2), Morocco (*n* = 1) and Panama (*n* = 2). It would have seemed reasonable to expect detailed series of triple infection cases from any Sub-Saharan country due to the high prevalence of HIV and TB coinfection, but we only found data from cohort studies, as we describe later.

As shown in Table 1, all cases except one were males, with an age range from 29 to 53 years. In addition, the clinical management and outcomes described for these patients could be considered similar to those with only COVID-19. The diagnosis of COVID-19 was coincident with the detection of the other infections in three out of the five cases. When HIV condition was previously known, there was not adherence to any antiretroviral therapy [38]. TB status was known in two patients, with a previous TB treatment including rifampicin and isoniazid for one of them [39], whereas no tuberculostatic was indicated for the other patient. When stated, the diagnosis of SARS-CoV-2 infection was performed by RT-PCR on nasopharyngeal exudates.

Regarding biochemical parameters assessment, three patients showed an elevation of C-reactive protein (CRP), the other three anemia and two of them a serum ferritin level above reference limits (Table 1). Lymphopenia was also found in all but one patient. Elevated CRP, lactate dehydrogenase and serum ferritin levels besides lymphopenia were previously established as predictors of COVID-19 severity and mortality (Table 1) [40,41,42]. However, respiratory support was needed in only half of cases [39]. Moreover, clinical management and therapy were not different from the ones used at early stage of the pandemic in the rest of countries: Hydroxychloroquine (HCQ) or chloroquine (CQ) associated with oral azithromycin. The potential use of azithromycin seemed to lay in its immunomodulatory effect as well as its ability to increase the effectiveness of HCQ in decreasing the viral load [43,44]. In addition, HCQ and CQ were proposed as treatment for COVID-19 also in the dawn of this pandemic, but recent clinical studies on COVID-19 patients treated with CQ/HCQ and AZM led to controversial results because of their inefficacy and adverse side effects [45,46,47]. All the patients in the cases series reviewed were completely recovered though (Supplementary File S4) [37,38,39].

Finally, we would like to remark the thromboprophylaxis treatment in Rivas and collaborators’ two cases with low molecular weight heparin [39]. COVID-19 severity and mortality were soon associated with an increment of prothrombotic parameters, particularly D-dimer levels [41,42]. Thus, anticoagulants were expected to be beneficial in mild and severe COVID-19 illness [48,49].

### 3.3. Narrative Review Findings

#### 3.3.1. General Overview

The coinfection of SARS-CoV-2 and *Mycobacterium tuberculosis* in HIV patients was highlighted as a matter of concern for some researchers [50]. We found models simulating transmission scenarios for HIV and TB, which predicted an increase of up to 10% and 20%, respectively, in high-burden settings compared with a non-COVID-19 situation [51]. However, reports describing cases which jointly captured these three concomitant infections, were anecdotal during last year, as already exposed in the previous section. Cohort studies were more abundant, such as an analysis carried out in Western Cape, South Africa, which showed an independent influence of HIV and TB on COVID-19 mortality [52].

Many authors appealed to elucidate the tangible influence of HIV and TB over COVID-19 severity, since resources were often scarce, with intensive care facilities completely full so the admission of patients had to be rationed [53]. This need of a thorough research was especially claimed for children, who represented a significant percentage of hospitalization in low- and middle-income countries, in particular sub-Saharan African countries [54]. Some COVID-19 surveillance studies and systems designed with the aim of determining the impact of HIV and TB on SARS-CoV-2 infection susceptibility, were ongoing or not finished yet [55].

Basic research focused on this triple infection was also found, showing that molecular studies evidenced an increased expression of angiotensin-converting enzyme 2 (ACE2), the SARS-CoV-2 entry receptor, in HIV/TB co-infected patients, but this finding was only a tissue level result [56]. Other authors pointed out a shared correlation between hijacking the endo-lysosomal system and the pathogenesis of the three microorganisms [57].

Despite the few targeted clinical and basic approaches retrieved in our literature search, the findings often spotlighted the social impact and public health consequences of the COVID-19 pandemic on the landscape of HIV and TB patients, so we have dedicated a specific section at the end of the Results section.

#### 3.3.2. Dual Infection Scenario (Either HIV or MTB with SARS-CoV-2)

Co-infection of SARS-CoV-2, either with HIV or MTB, was more common in literature. Regarding HIV co-infection, there existed an adequate evidence to challenge if PLWHIV were at higher risk of contracting SARS-CoV-2, as firstly speculated, or not. Some immunological features were shared by both viral infections. The acute phase of HIV infection was characterized by an important decline of CD4+ T cell count that persistently remained during chronic phase, leading to the well-known lymphopenia observed in untreated patients with AIDS. On the other hand, a poor clinical outcome of COVID-19 also related to lymphopenia due to the suppression of B lymphocytes, and helper (CD4+) and cytotoxic (CD8+) T cell disfunction [29,31,40,58,59].

In addition, HIV-positive persons usually presented comorbidities linked to risk factors for severity of COVID-19 symptoms, such as cardiovascular disease, diabetes and hypertension [60,61,62,63]. Smoking was also pointed out as a factor contributing to the higher prevalence and mortality in the current COVID-19 pandemic, and even in other infectious diseases, such as HIV and TB themselves [64]. Ageing of PLWHIV, as positive long-term result of ART, might become another risky feature for COVID-19 in these patients [40,65,66]. However, clinical case series reported worldwide during the earlier stage of the COVID-19 pandemic (first semester of 2020) showed a similar disease severity, with HIV patients admitted to intensive care and then discharged alive, in a comparable percentage of the general population [66,67,68,69,70,71,72,73,74,75,76,77]. This similar situation appeared even when HIV infection was not well controlled and patients presented a prior AIDS-defining event [72,78].

Furthermore, the so-called cytokine storm in patients with COVID-19 was related to severity, as already demonstrated in similar diseases, like SARS and Middle East respiratory syndrome (MERS) [79,80,81,82,83]. Thus, some researchers emphasized on ways to avoid the overproduction of these inflammatory mediators in order to reduce the damage and improve COVID-19 outcomes [82]. In that line, HIV infection was associated with a persistent immune disruption, even despite an effective ART, so such dysregulation could paradoxically prevent the mentioned cytokine release in severe and critical COVID-19 [84,85].

ART was also hypothesized as the reason of the lack of an increased risk for serious COVID-19 among HIV-positive persons. However, the findings were contradictory. HIV-positive patients receiving tenofovir-based ARTs were found to have a lower risk of hospitalization related to severe COVID-19 than those receiving other therapies [52,66], whereas other authors ruled out the possibility of a protective effect of tenofovir or any other antiretroviral therapy [70,78,86].

As the pandemic progressed, more data about HIV-positive patients became available and the equal risk perceived in HIV- and SARS-CoV-2 coinfected individuals was turning around a misperception. The key predictors for poor outcomes of COVID-19 mentioned (i.e., age and comorbidities) in the dual viral infection remained being the same ones as seen for SARS-CoV-2 exclusive infection [68,87,88]. Nevertheless, in the light of new research arose, advanced immunodeficiency and uncontrolled HIV-infection, previously underestimated as risk factors by some studies [72], were now well established as possible determinants of severity of COVID-19, showing increased rates of hospitalization and death [52,89,90]. Furthermore, lymphopenia in severe COVID-19 was prognostic of poor outcomes [26,42], so it would be logical to assume that immunodeficiency (defined as CD4 count <350/µL) during admission in PLWH was associated with COVID-19 death. Special mention to Hoffmann and collaborators’ study who did not find any significant association between severity of COVID-19 and a detectable HIV RNA, a prior AIDS-defining illness or TDF, as we mentioned before, but they did find it for a CD4+ T cell nadir of <200/µL and a current CD4+ T cells count <350/µL [78].

Boulle and collaborators performed a large-scale population cohort study in Western Cape, South Africa, finding a two-fold and almost a three-fold increased risk of mortality from COVID-19 for patients coinfected with HIV and active TB, respectively [52]. In PLWH, this greater risk was found even in patients aged younger than 50 years and independently of their viral suppression [52]. However, a high prevalence of comorbidities in deceased PLWH could be acting as a confounding factor [52]. Another study came to similar conclusions with regards to COVID-19 mortality and comorbidities in HIV and/or TB co-infected population: HIV, TB or both diseases were highlighted not to be the most common risk factors in comparison to older age and other comorbidities, such as diabetes and hypertension, in individuals dying from COVID-19 [91]. Nevertheless, HIV-infected individuals should not be considered to be protected from severe COVID-19. They must receive the same therapeutic approach applied to general population, and even be prioritized SARS-CoV-2 vaccination [77,90].

On the other hand, when considering TB-SARS-CoV-2 coinfection, it seemed evident that environmental conditions surrounding people who suffered most from TB were the best breeding ground for them to also be affected by the COVID-19 pandemic [92]. In addition, both TB and COVID-19 were respiratory infections, transmitted mainly by close contacts, and with similar symptoms. They might interplay reciprocally, but they were quite different: TB could be a chronic disease with patients coughing for a minimum of a couple of weeks, whereas COVID-19 had a quick onset. A differential diagnosis between both infections was possible and simple when healthcare providers were well-trained [34].

COVID-19 pneumonia may contribute to TB progression, whereas pulmonary TB impact the lungs’ function, so co-infection with SARS-CoV-2 might worsen COVID-19 prognosis itself. Tuberculosis could intensify COVID-19 by two hypothesized ways: impairing immune responses and increasing angiotensin converting enzyme 2 receptor expression in respiratory epithelial cells [58]. A meta-analysis performed by Tamuzi and collaborators found that TB was a risk factor for COVID-19 severity and even mortality, regardless HIV status [93]. However, case series did not report any contribution of COVID-19 to TB pathogenesis, so their reciprocal influence could not be excluded or confirmed [94,95].

When combining environmental and pathogenic issues, studies reported a higher expected probability of long-term effects of the pandemic in people with TB, TB-HIV coinfection or chronic lung disease [96]. A recent meta-analysis considering dual infection, either TB and HIV, showed an increased risk of mortality for the former, but a comparable clinical outcome of COVID-19 in patients with or without this latter infection [97]. Similarly, another study in Nigeria pointed to a higher mortality rate in states with high TB prevalence (with a weak association though), and the opposite finding for HIV infection: a negative correlation between HIV prevalence and COVID-19 mortality [98]. However, other reviews, even those aimed at children, did not find a significant risk factor for severe COVID-19 in pre-existing respiratory diseases [99]. Furthermore, our collection of specific case series after the literature review (Supplementary File S4), did not show any significant difference at clinical management or future scenario of healing. In four out of five cases, the clinical assessments to investigate COVID-19 facilitated the identification of pre-existing TB and/or HIV. HIV and TB co-infection was known but uncontrolled in only one case [38]. In addition, both infections were previously unidentified and simultaneously diagnosed in two out of all cases [37,39].

Nevertheless, the achievement of a considerable body of evidence for TB and HIV pre-existing conditions as determinants to predict COVID-19 progression would require the design of a research protocol a priori considering them, like Allwood and collaborators proposed [100]. Particular focus should be on how COVID-19 differently manifested among women with HIV and/or TB, endemic in Sub-Saharian African countries and so, a high infections burden setting [101].

#### 3.3.3. Social and Public Health Implications in the Context of the COVID-19 Pandemic

Most of our literature findings also warned about the impact of COVID-19 pandemic over the diagnosis, healthcare, drug supply, and even research about patients affected by any or both other two infections [102]. The current pandemic could lead to an amplification of the existing health inequities, especially concerning already marginalized communities, racial/ethnic minorities, women, and children [103,104,105,106]. The most dramatic influence would obviously be observed in low- and middle-income countries, already ravaged by TB and HIV epidemics [33,34,36]. When the majority of developed world was already immersed in COVID-19 vaccine rollout, some voices were pleading in favour of a global health equity and justice to achieve a factual end of this pandemic [107]. Vaccination in countries with a significant burden of the HIV and TB co-infections must be done guaranteeing safety and efficacy of available vaccines, besides affordable cost and manufacturing infrastructure. Addressing the first issue of safety, immunocompromised individuals should only be vaccinated with certain vaccine modalities, different from live-attenuated or replicating vaccines; regarding vaccine efficacy, the magnitude and durability of immunity in such patients were up to date unknown [108].

One of the first issues threatened by the pandemic was the diagnosis for both TB and HIV infections, since their diagnostic platforms, i.e., GeneXpert and PCR, were devoted to COVID-19 tests [33,109,110]. Furthermore, the rigid lockdowns enforced in many countries like South Africa, emulating the first Chinese mitigation strategies, also jeopardized HIV and TB care services [35,111]. So, a six-month disruption of ART in PLWHIV was estimated by WHO and other researchers to lead to a substantial excess of mortality due to AIDS-related conditions, such as TB itself [51,112]. Even though the drug supply was assured during the current COVID-19 pandemic, the fear of being exposed to SARS-CoV-2 infection or the difficult of commuting (many HIV and TB patients relied on public transportation) halted the normal access to health point of care or disrupted the routinely attendance to clinics [33,111,113].

At this time with three ongoing epidemics, some authors have suggested key adaptations to ensure delivery of healthcare services while reducing the risks of SARS-CoV-2 exposure in high HIV/TB burden settings. Take, for instance, any situation avoiding unnecessary visits, such as a common starting treatment day in HIV-TB co-infected patients, if any of the infections were diagnosed but not treated yet; providing sufficient TB and/or HIV treatment; or limiting in-person interactions with health care workers to clinical assessments at the completion of treatment or with urgent results (e.g., positive serum cryptococcal antigen test, drug resistances, etc.). Other consultations should be done telephonically [114].

Another dramatic consequence that the ongoing COVID-19 pandemic might cause over TB and HIV infections (and extensively over other infectious diseases) was represented by the delay and even the dropout of new approaches to fighting against drug resistance [102]. A successful completion of any treatment was compromised as we discussed before, with a special impact on pediatric age groups [104,115].

However, not all the measures to prevent COVID-19 had led to a deterioration of TB and HIV policies. For instance, wearing a surgical mask might reduce the transmission of TB [116]. In addition, we observed an acceleration of scientific and innovation approaches (new treatment, cutting-edge vaccines, etc.). However, some authors put the spotlight in global funding models and in international diplomacy in order to distribute those achievements equally, always avoiding the disruption of essential healthcare services [117,118].

## 4. Conclusions

We need more thoroughly observational and cohort studies whose design deliberately embraces the triple infection as a priori inclusion criterion in the target population. Only then, we will be able to confirm the influence on clinical evolution, management, therapy and outcomes of COVID-19 patients with HIV and TB infections. At the moment, the main concern is the continuity of all essential services for HIV/AIDS and TB patients. Most of them have been achieved after arduous effort, especially in many developing countries where they are currently and seriously being threatened.

## Figures and Tables

**Table 1:** Main characteristics of the 5 triple-infection cases reported.

Cases	Origin	Age (Years)	Gender	HIV and SARS-CoV-2 Coincident Diagnosis? (Yes/No)	AIDS-Defining Condition	TB and SARS-CoV-2 Coincident Diagnosis? (Yes/No)	Haematological Abnormalities	Biochemical Assessment	Exertional Dyspnea	Fever	Respiratory Therapy (Yes/No)	Reference
1	Morocco	32	Female	Yes	No	Yes	Thrombocytopenia and leucopenia	Anemia, hyper-ferritinemia	Yes	Yes	No	[37]
2	Brazil	43	Male	No ^a^	Yes	Yes	Lymphopenia, low haemoglobin and haematocrit levels	Increase of LDH and CRP	No	No	Not stated	[38]
3	Brazil	39	Male	No ^a^	Yes	No	Lymphopenia, low haemoglobin and haematocrit levels	Increase of CRP	Mild	Yes	No ^b^	[38]
4	Panama	53	Male	Yes	No	No	Not stated	Mild anemia	Moderate	Yes	Yes ^c^	[39]
5	Panama	29	Male	Yes	No	Yes	Neutrophilia and lymphopenia	Mild anemia, elevation of CRP, ferritin, D-dimer and procalcitonin	Moderate	No	Yes	[39]

Abbreviations: CRP: C-reactive protein; LDH: lactate dehydrogenase ^a^ Non-adherence to antiretroviral therapy ^b^ Just oxygen during first 3 days ^c^ Confirmed pneumonia.

## Supplementary Materials

The following are available online at https://www.mdpi.com/article/10.3390/v13050931/s1, Supplementary File S1: Publications retrieved in our literature search, Supplementary File S2: Studies discarded from the original literature search, Supplementary File S3: Studies that met the inclusion criteria, Supplementary File S4: Case_series: Case reports found.
